# Curcumin Precisely Regulates Ochratoxin A-Induced Apoptosis in Porcine Renal Epithelial Cells via the PI3K/AKT/mTOR Signaling Pathway

**DOI:** 10.3390/toxins18070315

**Published:** 2026-07-20

**Authors:** Yingyi Wu, Shuying Lin, Chuhan Shao, Cheng Zhang, Kaiyin Xie, Kaizhao Zhang, Xiaohong Huang, Hongjie Cui

**Affiliations:** 1 Fujian Key Laboratory of Traditional Chinese Veterinary Medicine and Animal Health, College of Animal Sciences, Fujian Agriculture and Forestry University, Fuzhou 350002, China; wu.yingyi2026@outlook.com (Y.W.);; 2 University Key Laboratory for Integrated Chinese Traditional and Western Veterinary Medicine and Animal Healthcare in Fujian Province, Fujian Agriculture and Forestry University, Fuzhou 350002, China

**Keywords:** curcumin, ochratoxin A, porcine renal epithelial cells, apoptosis, transcriptome sequencing, PI3K/AKT/mTOR signaling pathway

## Abstract

Ochratoxin A (OTA) is a widespread nephrotoxic mycotoxin that contaminates cereal grains and feed, posing serious threats to animal health and food safety. Although curcumin has been reported to exert protective biological activities, it remains unclear whether its protection against OTA-induced renal epithelial injury is mediated by broad transcriptomic reversal or by selective regulation of key survival pathways. This study investigated the protective effect of curcumin against OTA-induced apoptosis in porcine renal epithelial (PK-15) cells and explored the underlying molecular mechanism using transcriptome sequencing combined with molecular validation. PK-15 cells were treated with 8 μg/mL OTA (approximately 19.8 μmol/L), 10 μmol/L curcumin, or their combination. Cell viability, LDH release, apoptotic morphology, mitochondrial membrane potential (ΔΨm), and apoptosis rate were assessed by CCK-8, LDH assay, Hoechst 33342 staining, JC-1 staining, and flow cytometry. Transcriptome sequencing was performed to identify global gene expression changes and key signaling pathways, followed by qRT-PCR and Western blot validation of apoptosis-related factors and the PI3K/AKT/mTOR pathway. OTA markedly reduced cell viability, increased LDH release, induced nuclear condensation and apoptotic body formation, and decreased ΔΨm. Transcriptome analysis revealed that OTA caused extensive transcriptional dysregulation (11,707 differentially expressed genes), whereas curcumin selectively modulated 498 genes, of which 380 overlapped with OTA-responsive genes. KEGG enrichment identified the PI3K-Akt signaling pathway as a key regulatory target. At the mRNA level, OTA upregulated *Bax* and *Caspase-3* and downregulated *Bcl-2*; corresponding changes were observed at the protein level, and curcumin reversed these effects. Furthermore, OTA reduced the mRNA levels of *PI3K*, *AKT*, and *mTOR* and the protein abundance of PI3K, p-AKT, and mTOR, whereas curcumin partially restored these changes. In conclusion, curcumin alleviates OTA-induced mitochondrial apoptosis in PK-15 cells mainly by restoring PI3K/AKT/mTOR-associated survival signaling and Bcl-2/Bax/Caspase-3 balance rather than by broadly reversing the entire transcriptomic disturbance. These findings provide mechanistic insight into curcumin-mediated protection against OTA-induced renal epithelial toxicity.

## 1. Introduction

Ochratoxin A (OTA) is a secondary metabolite produced by fungi of the genera Aspergillus and Penicillium, and it commonly contaminates cereals, animal feed, and various food products [1,2]. On a global scale, OTA contamination is frequently detected in cereals and cereal-derived products.

A systematic review and meta-analysis based on global data collected between 2000 and 2023 revealed that the prevalence of OTA contamination in wheat and wheat flour reached as high as 53% [3]. Furthermore, OTA contamination has also been reported in feed ingredients such as soybean and soybean meal. A global survey encompassing 7049 samples demonstrated that the detection rate of OTA in soybean and soybean meal was approximately 28% [4].

As a potent nephrotoxin, OTA predominantly accumulates in renal tubular epithelial cells, where it induces tubular injury and renal dysfunction by inhibiting protein synthesis, promoting oxidative stress, and disrupting mitochondrial function [5,6].

OTA exposure is closely associated with human kidney disorders. Epidemiological studies and case–control investigations indicate that, in the Balkan region, OTA may act as a causative factor in the development of Balkan endemic nephropathy and is linked to an increased risk of urinary tract tumors [7].

Additionally, the ingestion of OTA-contaminated feed by livestock and poultry not only impairs production performance but also results in OTA residues accumulating in edible tissues, particularly the kidneys, thereby posing a potential threat to human health through food chain transmission [8].

The mechanisms underlying OTA-induced renal cell apoptosis involve multiple signaling pathways. Previous studies have demonstrated that OTA can disrupt mitochondrial membrane potential and activate the caspase cascade, thereby triggering the intrinsic apoptotic pathway [9,10]. In addition, the PI3K/AKT/mTOR signaling pathway serves as a central regulator of cell survival, metabolism, and anti-apoptotic processes, and plays a pivotal role in toxin-induced cellular injury [11,12]. Inactivation of this pathway can disrupt the balance of the Bcl-2 family proteins, characterized by the downregulation of Bcl-2 and upregulation of Bax, thereby promoting mitochondrial pathway-mediated apoptosis [13]. However, direct evidence is still lacking to confirm whether OTA initiates the apoptotic program by inhibiting the PI3K/AKT/mTOR pathway.

Curcumin is a natural polyphenolic compound extracted from the rhizome of turmeric, attracting considerable attention because of its low toxicity and multi-target pharmacological activities [14,15]. Previous studies have demonstrated that curcumin exerts anti-inflammatory, antioxidant, and anti-apoptotic effects in various models of hepatic and renal injury by scavenging free radicals and modulating the NF-κB and Nrf2 signaling pathways [16,17,18]. Notably, curcumin exhibits a potential protective effect against OTA-induced renal injury [19,20]. However, the precise molecular mechanisms, particularly the global regulatory patterns at the transcriptomic level, remain unclear. It is also unclear whether curcumin protects renal epithelial cells by broadly reversing OTA-induced transcriptional dysregulation or by selectively regulating a limited number of key survival pathways. Clarifying this issue may help define how natural products counteract mycotoxin-induced renal injury.

In this study, porcine kidney epithelial cells (PK-15) were employed as an in vitro renal epithelial model because the kidney is the major target organ of OTA toxicity and PK-15 cells provide a reproducible porcine-origin system for evaluating OTA-induced epithelial injury. Although this cell line cannot fully reproduce the complexity of in vivo nephrotoxicity, including renal hemodynamics, intercellular communication, metabolism, and immune responses, it is suitable for mechanistic screening of mitochondrial apoptosis and signaling pathway regulation. A combination of cell viability assays, apoptotic phenotyping, transcriptome sequencing, and molecular biology validation was applied. Notably, an unbiased transcriptomic approach was employed to compare global gene expression profiles between OTA-exposed and curcumin-treated cells, with the aim of identifying key target pathways of curcumin and clarifying the involvement of the PI3K/AKT/mTOR signaling axis.

## 2. Results

### 2.1. Curcumin Alleviates OTA-Induced Cell Viability Inhibition and Cell Membrane Damage in PK-15 Cells

**Figure 1:**
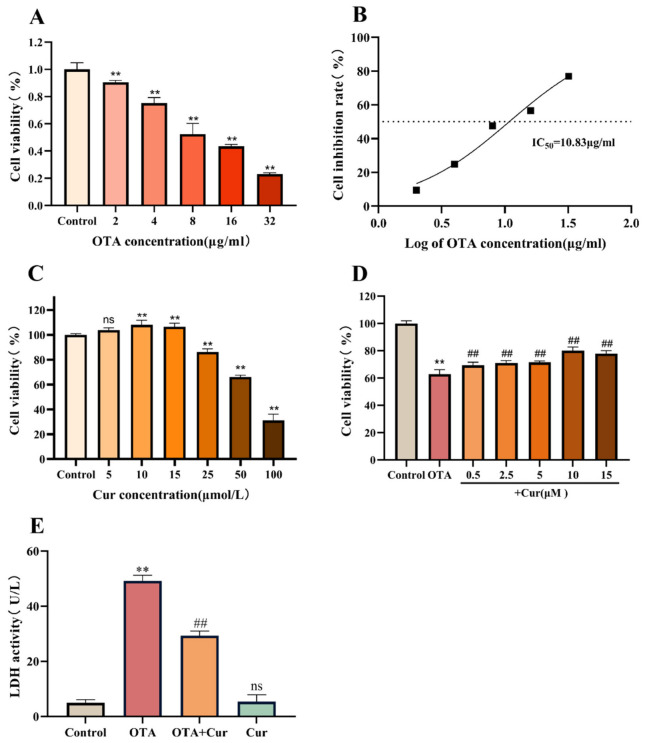
Effects of curcumin on OTA-induced cell viability reduction and LDH release in PK-15 cells. (**A**) CCK-8 assay showing PK-15 cell viability after 24 h treatment with increasing concentrations of OTA (0–32 μg/mL). (**B**) The IC_50_ of OTA was calculated as 10.83 μg/mL by non-linear regression; the dotted horizontal line marks 50% inhibition used to determine the IC_50_. (**C**) Cell viability after 24 h treatment with increasing concentrations of curcumin (0–100 μmol/L). (**D**) Cell viability in the Control and OTA groups and after co-treatment with OTA plus increasing curcumin concentrations (0.5–15 μmol/L). (**E**) LDH release in the culture supernatant of each group. Data are presented as mean ± SD (*n* = 3). ** *p* < 0.01 vs. Control group; ## *p* < 0.01 vs. OTA group; ns, not significant to Control group.

Cell viability was assessed by CCK-8 assay. The results demonstrated that OTA inhibited cell viability in a concentration-dependent manner, and OTA at concentrations above 8 μg/mL reduced cell viability to below 50% (Figure 1A). The half-maximal inhibitory concentration (IC_50_) of OTA was calculated by non-linear regression to be 10.83 μg/mL (approximately 26.8 μmol/L) (Figure 1B); therefore, 8 μg/mL OTA (approximately 19.8 μmol/L) was selected as a sub-IC_50_ injury dose for subsequent experiments. Curcumin at concentrations below 15 μmol/L demonstrated no obvious cytotoxicity (Figure 1C); therefore, 10 μmol/L curcumin was selected as a non-cytotoxic intervention concentration. These concentrations should be interpreted as in vitro exposure levels employed to establish a reproducible injury-protection model rather than direct physiological plasma concentrations.

LDH is a soluble cytoplasmic enzyme that is released into the culture medium when plasma membrane integrity is compromised. Therefore, LDH activity in the culture supernatant was used as an indirect indicator of membrane damage and cytotoxicity [21]. Compared with the control group, the OTA group demonstrated a highly significant decrease in cell viability (Figure 1D) and a highly significant increase in LDH release (Figure 1E), indicating impaired membrane integrity. Compared with the OTA group, the OTA+Cur group displayed markedly restored cell viability (Figure 1D) and reduced LDH release (Figure 1E), suggesting that curcumin alleviates OTA-induced cytotoxicity.

### 2.2. Curcumin Reduces OTA-Induced Apoptosis and Mitochondrial Membrane Potential Loss

**Figure 2:**
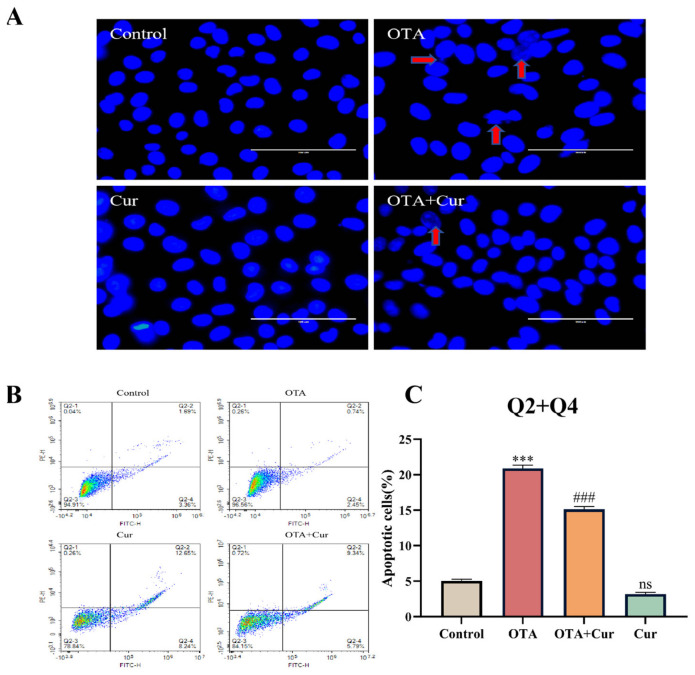
Curcumin reduces OTA-induced apoptosis in PK-15 cells. (**A**) Representative Hoechst 33342 staining images showing nuclear condensation and apoptotic bodies (arrows) in each group (scale bar = 100 μm). (**B**) Flow cytometry dot plots of Annexin V-FITC/PI staining; the PI fluorescence signal was recorded in the PE-H channel by the flow cytometer. Pseudocolor indicates event density, and the black quadrant lines indicate gating boundaries. (**C**) Quantitative analysis of the total apoptosis rate (early + late apoptosis) from three independent experiments. Data are presented as mean ± SD (*n* = 3). *** *p* < 0.001 vs. Control group; ### *p* < 0.001 vs. OTA group; ns, not significant to Control group.

Hoechst 33342 is a blue fluorescent dye that crosses the cell membrane. In apoptotic cells, the nuclei show condensed chromatin or fragmented apoptotic bodies [22]. Hoechst 33342 staining was used here as a qualitative morphological observation rather than as an independent quantitative or fully discriminatory assay. The OTA group demonstrated many condensed nuclei and apoptotic bodies, whereas these features were markedly reduced in the OTA+Cur group (Figure 2A). Flow cytometry was further used for quantitative assessment and confirmed that curcumin reduced OTA-induced apoptosis (Figure 2B,C).

**Figure 3:**
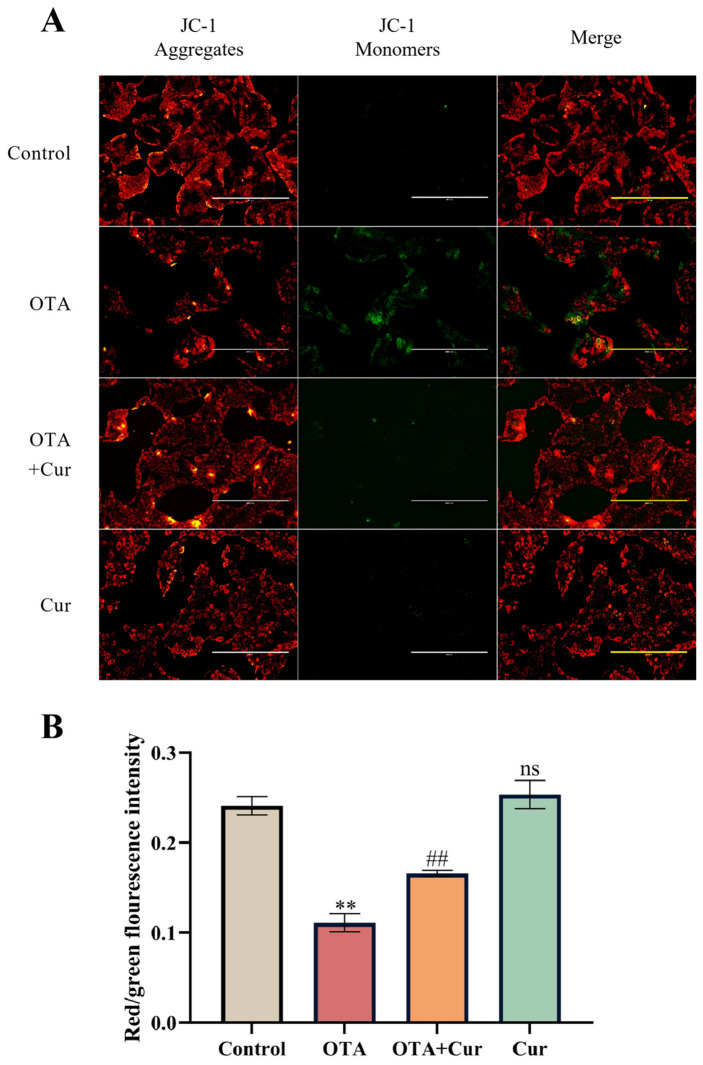
Curcumin attenuates OTA-induced loss of mitochondrial membrane potential (ΔΨm) in PK-15 cells. (**A**) Representative JC-1 staining images showing red fluorescence (aggregates, high ΔΨm) and green fluorescence (monomers, low ΔΨm) in each group (scale bar = 100 μm). (**B**) Quantification of the red/green fluorescence intensity ratio, reflecting mitochondrial membrane potential (ΔΨm). At least six randomly selected fields per group were analyzed for fluorescence quantification. Data are presented as mean ± SD (*n* = 3). ** *p* < 0.01 vs. Control group; ## *p* < 0.01 vs. OTA group; ns, not significant to Control group.

Loss of mitochondrial membrane potential (ΔΨm) is one of the earliest events in the apoptotic cascade. Once ΔΨm collapses, apoptosis becomes irreversible [23]. JC-1 is a mitochondrial fluorescent dye. In non-apoptotic cells with high ΔΨm, JC-1 forms J-aggregates in the mitochondrial matrix (red fluorescence); in apoptotic cells with low ΔΨm, JC-1 remains as monomers in the cytoplasm (green fluorescence). Thus, changes in ΔΨm can be reflected by the red/green fluorescence ratio [24,25,26]. JC-1 assay demonstrated that OTA treatment caused a decrease in red fluorescence and an increase in green fluorescence, resulting in a highly significant reduction in the red/green ratio (*p* < 0.01), indicating a decrease in ΔΨm. Curcumin intervention partially restored the red/green ratio (Figure 3A,B).

### 2.3. Transcriptome Sequencing Reveals Global Gene Expression Characteristics of OTA and Curcumin Intervention

To reveal the mechanisms of OTA-induced injury and the protective effect of curcumin in PK-15 cells, transcriptome sequencing was performed on the Control, OTA, and OTA+Cur groups.

**Figure 4:**
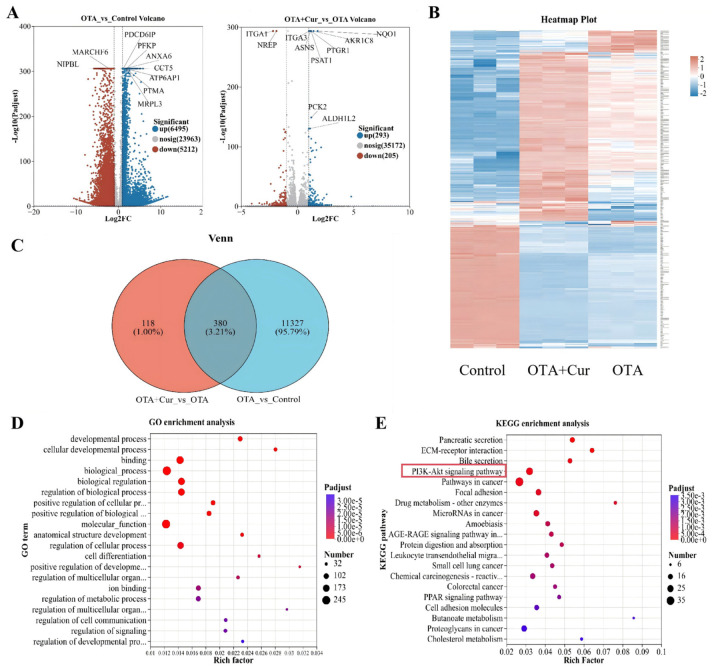
Transcriptome profiling of PK-15 cells in response to OTA and curcumin. (**A**) Volcano plots of differentially expressed genes (DEGs) in OTA_vs_Control (**left**) and OTA+Cur_vs_OTA (**right**). Red: up-regulated; blue: down-regulated (|log_2_FC| ≥ 1, FDR < 0.05). (**B**) Cluster heatmap of DEGs across Control, OTA, and OTA+Cur groups. Each column represents a sample, and each row represents a gene. Red indicates high expression, blue indicates low expression. (**C**) Venn diagram showing the overlap of DEGs between OTA_vs_Control and OTA+Cur_vs_OTA comparisons. (**D**) GO functional enrichment analysis of the 380 common DEGs. (**E**) KEGG pathway enrichment analysis of the 380 common DEGs. The size of the dots represents the number of enriched genes, and the color represents the adjusted *p*-value. The red box highlights the PI3K–Akt signaling pathway. Ellipses in panels D and E indicate labels truncated by the plotting software for display; the enrichment categories and their interpretation are described in the main text.

Differential expression analysis (Figure 4A,C) demonstrated that, relative to the control group, OTA treatment caused extensive transcriptional dysregulation, with a total of 11,707 differentially expressed genes (DEGs; 6495 up-regulated, 5212 down-regulated). In contrast, relative to the OTA group, the OTA+Cur group had only 498 DEGs (293 up-regulated, 205 down-regulated), accounting for approximately 4.25% of the OTA-induced DEGs. A total of 380 common DEGs were shared between the two comparisons. These data indicate that OTA exerts a global impact on the transcriptome, whereas curcumin selectively regulates a small number of key genes rather than broadly reversing all changes. The cluster heatmap (Figure 4B) demonstrated that the expression profile of the OTA+Cur group was intermediate between the Control and OTA groups, consistent with the partial phenotypic reversal.

GO enrichment analysis (Figure 4D) demonstrated that the common DEGs were mainly enriched in biological regulation, regulation of cellular processes, regulation of signaling, and other terms, suggesting that curcumin exerts its protective effect by restoring the homeostasis of cellular regulatory networks.

KEGG pathway enrichment analysis (Figure 4E) revealed that the common DEGs were markedly enriched in the PI3K-Akt signaling pathway, ECM–receptor interaction, and focal adhesion. The PI3K-Akt pathway is a classical central pathway regulating cell survival, proliferation, metabolism, and anti-apoptosis. Numerous studies have confirmed that activation of the PI3K/AKT pathway can phosphorylate downstream molecules such as Bad and Caspase-9, thereby inhibiting the mitochondrial apoptotic pathway. In this study, enrichment of the PI3K-Akt pathway suggests that OTA may promote apoptosis partly by suppressing this survival pathway, and that the protective effect of curcumin may be associated with partial reactivation of this pathway. Because several pathways were enriched, PI3K/AKT/mTOR should be interpreted as a key regulatory axis rather than the only pathway involved.

### 2.4. Curcumin Suppresses OTA-Activated Apoptotic Signaling Pathway

**Figure 5:**
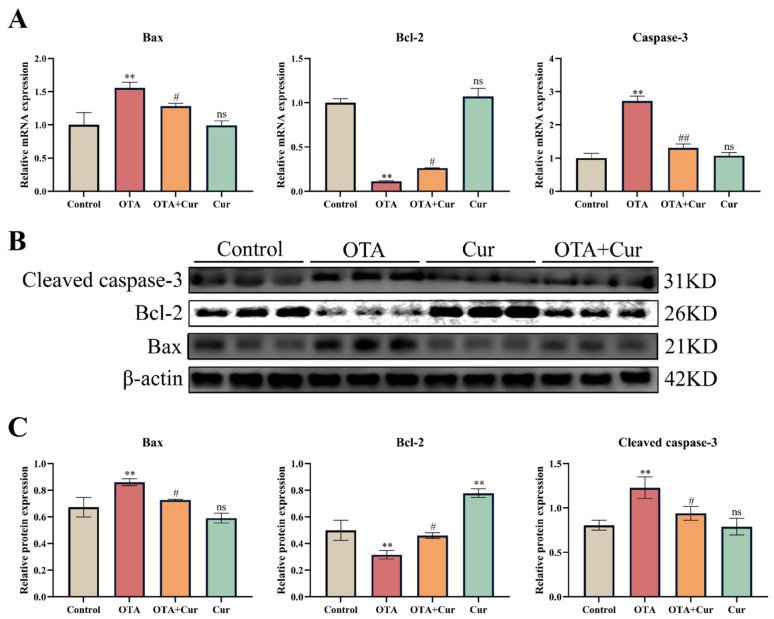
Curcumin suppresses OTA-activated apoptotic signaling pathway in PK-15 cells. (**A**) Relative mRNA expression levels of *Bax*, *Bcl-2*, and *Caspase-3* determined by qRT-PCR. *β-actin* was used as an internal control. (**B**) Representative Western blot bands of Bax, Bcl-2, and cleaved caspase-3. (**C**) Densitometric analysis of the protein levels normalized to β-actin. Data are presented as mean ± SD (*n* = 3). ** *p* < 0.01 vs. Control group; # *p* < 0.05 and ## *p* < 0.01 vs. OTA group; ns, not significant to Control group.

Based on the above phenotypic and transcriptomic clues, qRT-PCR was performed to further validate and quantitatively evaluate the regulatory effect of curcumin. qRT-PCR results (Figure 5A) demonstrated that, relative to the control group, the OTA group exhibited highly significant increases in the mRNA expression of the pro-apoptotic genes *Bax* and *Caspase-3* (*p* < 0.01), and a highly significant decrease in the anti-apoptotic gene *Bcl-2* (*p* < 0.01). Compared with the OTA group, the OTA+Cur group markedly reversed these changes (*p* < 0.05 or *p* < 0.01).

Changes in mRNA levels do not necessarily reflect functional protein expression or activation status, especially for Caspase-3, which must be cleaved into cleaved caspase-3 to execute apoptosis. Therefore, Western blot was further performed to detect the protein expression levels of Bax, Bcl-2, and the activated form cleaved caspase-3. Western blot results (Figure 5B,C) demonstrated that, relative to the control group, the OTA group had markedly increased protein expression of cleaved caspase-3 and Bax (*p* < 0.01), and markedly decreased Bcl-2 protein expression (*p* < 0.01), suggesting that OTA induces apoptosis in PK-15 cells. Compared with the OTA group, the OTA+Cur group demonstrated markedly decreased cleaved caspase-3 and Bax protein expression (*p* < 0.05), and markedly increased Bcl-2 protein expression (*p* < 0.05). These findings verify that OTA disrupts the intracellular apoptosis/survival balance by up-regulating pro-apoptotic genes and down-regulating anti-apoptotic genes, thereby initiating the apoptotic program, and that curcumin antagonizes OTA-induced apoptotic signals.

### 2.5. Curcumin Reactivates the OTA-Suppressed PI3K/AKT/mTOR Signaling Pathway

KEGG enrichment analysis of the transcriptome sequencing suggested that curcumin may exert its protective effect partly through the PI3K/AKT/mTOR pathway, thereby regulating downstream apoptotic factors. To further examine the involvement of this pathway, the mRNA and protein expression levels of core molecules in the PI3K/AKT/mTOR pathway were examined.

**Figure 6:**
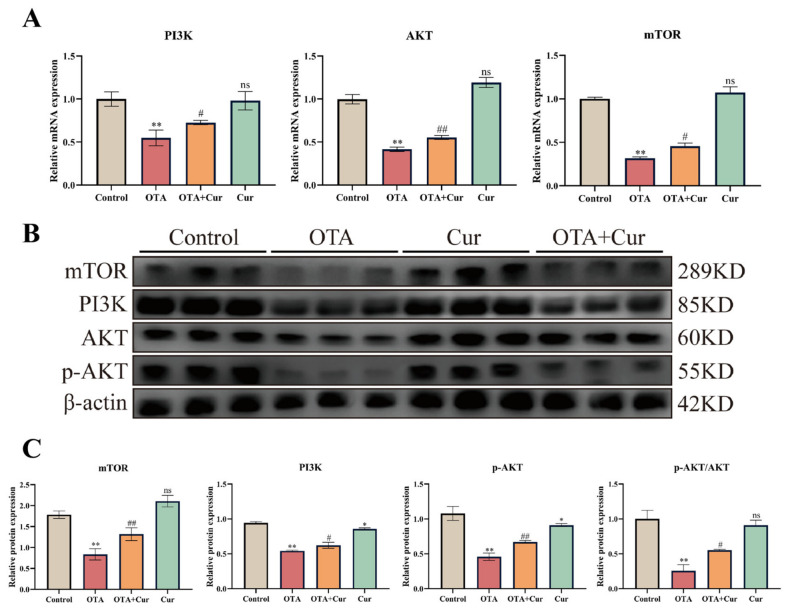
Curcumin reactivates the PI3K/AKT/mTOR signaling pathway suppressed by OTA in PK-15 cells. (**A**) Relative mRNA expression levels of *PI3K*, *AKT*, and *mTOR* determined by qRT-PCR. *β-actin* was used as an internal control. (**B**) Representative Western blot bands of PI3K, p-AKT, total AKT, and mTOR. (**C**) Quantitative analysis of protein expression (PI3K, p-AKT, mTOR) normalized to β-actin (p-AKT also normalized to total AKT). Data are presented as mean ± SD (*n* = 3). * *p* < 0.05 and ** *p* < 0.01 vs. Control group; # *p* < 0.05 and ## *p* < 0.01 vs. OTA group; ns, not significant to Control group.

qRT-PCR results (Figure 6A) demonstrated that the mRNA levels of *PI3K*, *AKT*, and *mTOR* in the OTA group were markedly lower than those in the control group (*p* < 0.01); curcumin treatment markedly restored the expression of these genes. Western blot results (Figure 6B,C) further demonstrated that OTA treatment reduced the protein levels of PI3K, p-AKT, and mTOR, while curcumin intervention markedly increased the expression of these proteins. These protein-level results are consistent with the qRT-PCR mRNA trends and support the involvement of PI3K/AKT/mTOR signaling in the protective effect of curcumin against OTA-induced apoptosis.

## 3. Discussion

This work provides, to our knowledge, a systematic elucidation at the phenotypic, transcriptomic, and molecular levels of the mechanisms by which curcumin antagonizes OTA-induced apoptosis in porcine kidney epithelial cells. Our key findings are as follows: (1) OTA treatment induced typical mitochondrial pathway-mediated apoptosis in PK-15 cells, manifested by decreased cell viability, elevated LDH leakage, collapse of mitochondrial membrane potential, and dysregulation of Bcl-2/Bax/Caspase-3 signaling; (2) transcriptome sequencing revealed that OTA disrupted the expression of over 11,700 genes, whereas curcumin selectively modulated fewer than 500 genes, indicating a targeted regulatory pattern rather than a global reversal effect; and (3) functional enrichment analysis identified the PI3K/AKT/mTOR pathway as a key mediator associated with curcumin’s action, and subsequent validation demonstrated that curcumin restored the OTA-suppressed activity of this pathway and rebalanced downstream apoptotic effectors. It should also be noted that OTA may induce necrosis or other forms of cell death at relatively high concentrations; therefore, the present study focuses mainly on apoptosis-associated mechanisms rather than fully excluding necrotic injury.

Previous studies have often focused on a limited set of OTA-responsive apoptosis-related genes [27]. The present work used transcriptomics to depict a broader landscape of transcriptional dysregulation in PK-15 cells under OTA exposure. The 11,707 differentially expressed genes spanned multiple biological processes, including cell signaling, metabolism, adhesion, and stress responses. This finding suggests that OTA nephrotoxicity is not a simple single-target effect but involves systematic disruption of cellular homeostatic networks. In contrast, curcumin intervention altered only 498 genes, accounting for 4.25% of the OTA-induced differentially expressed genes. This asymmetric outcome suggests that curcumin is not a nonspecific universal antagonist but may restore cellular homeostasis by modulating a limited number of critical regulatory nodes, particularly those related to cell survival and apoptosis [28,29].

Our data show that OTA markedly reduced the mRNA levels of *PI3K*, *AKT*, and *mTOR* as well as the protein abundance of PI3K, p-AKT, and mTOR; curcumin treatment partially restored these changes. Changes in downstream apoptotic regulators were consistent with the activity status of this pathway: OTA-induced inhibition of PI3K/AKT/mTOR was accompanied by decreased Bcl-2, increased Bax, and activation of Caspase-3, whereas curcumin treatment reversed these apoptotic signals. This observation is consistent with established regulatory mechanisms of the PI3K/AKT signaling axis on the Bcl-2 family, whereby AKT may regulate Bcl-2 and Bax through phosphorylation of Bad, inhibition of FoxO transcriptional activity, and activation of NF-κB [30]. Therefore, we propose that curcumin-mediated restoration of PI3K/AKT/mTOR signaling contributes to the recovery of Bcl-2/Bax balance and stabilization of mitochondrial membrane potential. However, this conclusion should be interpreted as pathway association and experimental support rather than definitive causality, because pathway inhibition or gene knockdown experiments were not performed in the present study.

Previous reports have indicated that curcumin can attenuate OTA-induced renal injury in rats, as evidenced by reductions in blood urea nitrogen and creatinine levels, as well as alleviation of histopathological alterations [19]. Other studies have shown that curcumin regulates apoptosis, autophagy, oxidative stress, and inflammatory pathways in different injury models [31,32]. Compared with these reports, the major novelty of the present study lies in the integration of transcriptomic analysis with cellular phenotypes and molecular validation, which allowed us to distinguish broad OTA-induced transcriptional disruption from the more selective curcumin-responsive gene set. In addition to PI3K/AKT/mTOR signaling, significant enrichment of ECM–receptor interaction and focal adhesion pathways was observed, suggesting that curcumin may also contribute to the maintenance of cytoskeletal integrity and cell–matrix adhesion. Therefore, PI3K/AKT/mTOR is highlighted as a key protective pathway, but not as the exclusive mechanism.

This study has several limitations. First, all experiments were conducted in PK-15 cells, and validation in in vivo animal models is lacking. The kidney is a complex organ in which intercellular interactions, hemodynamics, immune responses, metabolism, and systemic bioavailability may influence the actual protective effects of curcumin. Future studies should evaluate curcumin in OTA-intoxicated rodent or piglet models and measure physiological, biochemical, and histopathological endpoints, including body weight, kidney index, serum creatinine, blood urea nitrogen, urinary injury biomarkers, OTA residues, renal histopathology, tubular injury scores, oxidative stress markers, and apoptosis-related proteins. Second, curcumin has limited bioavailability and stability, which may restrict direct practical application. Formulation strategies such as nanoemulsions, liposomes, micelles, phospholipid complexes, or other delivery systems should be considered to improve its effectiveness. Third, although transcriptomic and protein-level analyses highlighted PI3K/AKT/mTOR signaling, causal verification using specific pathway inhibitors (e.g., LY294002 or MK-2206) or gene knockdown approaches was not performed. Fourth, oxidative stress markers such as ROS, SOD, CAT, GPx, MDA, or Nrf2 signaling were not measured, although oxidative stress is a major mechanism of OTA toxicity and curcumin activity. Fifth, the validation of mitochondrial apoptosis focused on Bax, Bcl-2, and Caspase-3; upstream markers such as Cytochrome c and Caspase-9 should be included in future studies. Additionally, OTA may trigger necrosis, ferroptosis, and autophagic cell death [33,34,35], and the potential crosstalk between these cell death pathways warrants further investigation.

## 4. Conclusions

In summary, OTA disrupts mitochondrial homeostasis and induces apoptosis in PK-15 renal epithelial cells, at least in part, through suppression of the PI3K/AKT/mTOR signaling pathway. Curcumin mitigates OTA-induced cytotoxicity by restoring this survival pathway and rebalancing downstream Bcl-2/Bax/Caspase-3 apoptotic signaling. Rather than broadly reversing all OTA-induced transcriptomic alterations, curcumin appears to exert selective regulatory effects on key signaling nodes. These findings provide mechanistic insight into curcumin-mediated cytoprotection against OTA-induced renal epithelial toxicity and support further investigation of curcumin or optimized curcumin formulations in in vivo models. Nevertheless, practical application requires additional validation considering curcumin bioavailability, dose–response relationships, long-term safety, and systemic complexity.

## 5. Materials and Methods

### 5.1. Cell Culture and Treatment

Porcine renal epithelial PK-15 cells were obtained from the American Type Culture Collection (ATCC, Manassas, VA, USA). Cells were cultured in Dulbecco’s Modified Eagle Medium (DMEM, high glucose, HyClone, Logan, UT, USA) supplemented with 10% fetal bovine serum (HyClone) and 1% penicillin–streptomycin (HyClone) in a humidified incubator at 37 °C with 5% CO_2_. Ochratoxin A (OTA, purity ≥ 98%, molecular weight 403.81 g/mol, Acmec Biochemical Co., Ltd., Shanghai, China) and curcumin (Cur, purity ≥ 94%, Sigma-Aldrich, St. Louis, MO, USA) were dissolved in dimethyl sulfoxide (DMSO, Sigma-Aldrich) and further diluted in culture medium. For unit comparison, 8 μg/mL OTA corresponds to approximately 19.8 μmol/L. The final DMSO concentration in all treatment groups never exceeded 0.1% (*v*/*v*), which was previously confirmed to have no effect on cell viability.

For experiments, cells were grown to 70–80% confluence and then treated as follows: (1) Control (culture medium only), (2) OTA group (8 μg/mL OTA, approximately 19.8 μmol/L), (3) Cur group (10 μmol/L curcumin), and (4) OTA+Cur group (8 μg/mL OTA + 10 μmol/L curcumin). The OTA concentration was selected as a sub-IC_50_ injury dose, while the curcumin concentration was selected as a non-cytotoxic intervention dose based on the preliminary viability assays. All treatments were performed for 24 h unless otherwise stated.

### 5.2. Cell Viability Assay (CCK-8)

Cell viability was assessed using the Cell Counting Kit-8 (CCK-8, Sigma-Aldrich). PK-15 cells were seeded into 96-well plates at 1 × 10^5^ cells/well and allowed to adhere for 24 h. For concentration–response experiments, cells were treated with various concentrations of OTA (0, 2, 4, 8, 16, 32 μg/mL) or curcumin (0, 5, 10, 15, 25, 50, 100 μmol/L) for 24 h. For the protective effect, cells were pre-treated with 10 μmol/L curcumin for 2 h, followed by co-incubation with 8 μg/mL OTA for 24 h. After treatment, cells were gently washed with PBS to remove residual compounds before the CCK-8 reaction. Then, 10 μL of CCK-8 solution was added to each well, and the plates were incubated for 2 h at 37 °C in the dark. Absorbance was read at 450 nm using a microplate reader (SpectraMax Mini, Molecular Devices, San Jose, CA, USA). Cell viability (%) = (absorbance of treated group/absorbance of control group) × 100. The half-maximal inhibitory concentration (IC_50_) of OTA was calculated by non-linear regression using GraphPad Prism 9.0 (GraphPad Software, San Diego, CA, USA). All experiments were performed in triplicate and repeated three times.

### 5.3. Lactate Dehydrogenase (LDH) Release Assay

Cell membrane integrity was evaluated by measuring LDH release into the culture medium using an LDH assay kit (Nanjing Jiancheng Bioengineering Institute, Nanjing, China). PK-15 cells were seeded into 6-well plates at 7 × 10^4^ cells/well and treated as described in Section 5.1. After 24 h, the culture supernatant was collected and centrifuged at 2500× *g* for 10 min at 4 °C to remove debris. LDH activity in the supernatant was measured according to the manufacturer’s protocol. Absorbance was read at 490 nm using a microplate reader. LDH release was expressed as a percentage relative to the control group. Each treatment was performed in six technical replicates, and the experiment was independently repeated three times.

### 5.4. Hoechst 33342 Staining for Nuclear Apoptotic Morphology

Nuclear morphological changes characteristic of apoptosis were visualized using Hoechst 33342 (Beyotime Biotechnology, Shanghai, China). After treatment, cells were stained with Hoechst 33342 at a final concentration of 10 μg/mL for 15 min at 37 °C in the dark, and then washed twice with phosphate-buffered saline (PBS, HyClone). Fluorescence images were captured using a fluorescence microscope (Thermo Fisher Scientific, Waltham, MA, USA) with excitation at 350 nm and emission at 460 nm. Cells exhibiting condensed chromatin and fragmented nuclei (bright blue fluorescence) were identified as apoptotic. Because Hoechst 33342 staining is mainly a morphological and semi-quantitative method, it was used only as supportive evidence of apoptotic morphology, while apoptosis was quantitatively evaluated by flow cytometry. At least six randomly selected fields per group were examined, and representative images are shown.

### 5.5. Flow Cytometric Analysis of Apoptosis

Apoptosis was quantitatively analyzed using the Annexin V-FITC/PI double-staining kit (Beyotime Biotechnology). After treatment, PK-15 cells were harvested by trypsinization without EDTA, washed twice with cold PBS, and resuspended in 1× Annexin V binding buffer at a density of 1 × 10^6^ cells/mL. Then, 100 μL of cell suspension was incubated with 5 μL of Annexin V-FITC and 5 μL of propidium iodide (PI) for 15 min at room temperature in the dark. After adding 400 μL of binding buffer, samples were analyzed within 1 h using a flow cytometer (BD FACSCanto™ II, BD Biosciences, Franklin Lakes, NJ, USA). Annexin V-FITC was detected in the FITC channel, and the PI signal was recorded in the PE-H channel according to the instrument configuration. A minimum of 10,000 events were acquired per sample. Data were analyzed with FlowJo software (version 10.6, BD Biosciences). The total apoptosis rate was calculated as the sum of early apoptotic (Annexin V^+^/PI^−^) and late apoptotic (Annexin V^+^/PI^+^) cells.

### 5.6. Mitochondrial Membrane Potential (ΔΨm) Measurement

Mitochondrial membrane potential was measured using the JC-1 probe (Zhuocai Biotechnology Co., Ltd., Shanghai, China). PK-15 cells seeded in 6-well plates were treated as described in Section 5.1 for 24 h. After removal of the culture medium, cells were washed once with PBS and then incubated with 1 mL of JC-1 staining working solution for 20 min at 37 °C in the dark. The cells were washed twice with JC-1 buffer (1×) and overlaid with 2 mL of culture medium. Fluorescence images were captured with a fluorescence microscope (Thermo Fisher Scientific) using excitation/emission at 488 nm/525 nm for the monomeric form (green, low ΔΨm) and at 543 nm/590 nm for the aggregated form (red, high ΔΨm). At least six randomly selected fields per group were analyzed, and fluorescence intensity was quantified using ImageJ software version 1.54t (National Institutes of Health, Bethesda, MD, USA; https://imagej.nih.gov/ij/; accessed on 14 July 2026). ΔΨm was expressed as the red/green fluorescence ratio.

### 5.7. Transcriptome Sequencing (RNA-Seq)

Total RNA was extracted from PK-15 cells of the Control, OTA, and OTA+Cur groups using TRIzol reagent (Invitrogen, Carlsbad, CA, USA). RNA quality and quantity were assessed, and qualified samples were sent to Shanghai Majorbio Bio-pharm Technology Co., Ltd. (Shanghai, China) for transcriptome sequencing on an Illumina NovaSeq 6000 platform (Illumina, San Diego, CA, USA). The sequencing data were analyzed online using the Majorbio Cloud Platform (https://www.majorbio.com; accessed on 14 July 2026). Briefly, differentially expressed genes (DEGs) were identified with the criteria |log_2_ fold change| ≥ 1 and FDR < 0.05. GO functional enrichment and KEGG pathway enrichment analyses were performed on the common DEGs.

### 5.8. Quantitative Real-Time PCR (qRT-PCR)

Total RNA was extracted using a kit (Promega, Beijing, China), and qRT-PCR was performed with TB Green Premix Ex Taq™ II (TaKaRa Bio Inc., Shiga, Japan) on a QuantStudio™ 5 system (Thermo Fisher Scientific, Waltham, MA, USA). Relative mRNA expression was calculated using the 2^−ΔΔCt^ method with *β-actin* as internal control. Primers are listed in Table 1.

### 5.9. Western Blot Analysis

Cells were lysed in RIPA buffer with protease/phosphatase inhibitors (Roche Diagnostics GmbH, Mannheim, Germany). Protein concentrations were determined by BCA kit (Thermo Fisher Scientific, Waltham, MA, USA). Equal amounts (30 μg) were separated by SDS-PAGE and transferred to PVDF membranes. After blocking with 5% non-fat milk, membranes were incubated overnight at 4 °C with primary antibodies (all from Cell Signaling Technology, Danvers, MA, USA): anti-Bax, anti-Bcl-2, anti-cleaved caspase-3, anti-PI3K, anti-p-AKT, anti-total AKT, anti-mTOR, and anti-β-actin (1:1000). HRP-conjugated secondary antibody (1:2000) was then applied. Blots were imaged using ECL and a ChemiDoc system (Bio-Rad Laboratories, Hercules, CA, USA); linear-range exposures were quantified in ImageJ after background subtraction. p-AKT was normalized to total AKT; other proteins were normalized to β-actin. Experiments were repeated three times.

### 5.10. Statistical Analysis

Data are expressed as mean ± SD from at least three independent experiments. Statistical comparisons were performed using one-way ANOVA with Tukey’s post hoc test or two-tailed Student’s t-test using IBM SPSS Statistics v25 (IBM Corp., Armonk, NY, USA). A *p*-value < 0.05 was considered significant. Graphs were generated with GraphPad Prism 9.0.

## Figures and Tables

**Table 1:** Primer sequences used for qRT-PCR.

Gene	Accession No.	Primer Sequence (5′→3′)	Product Size (bp)
*Bax*	XM_003127290.5	F: GCCGAAATGTTTGCTGACG R: GCAGCCGAGATAGAAGGA	157
*Bcl2*	NM_214285.1	F: GAGCGTAGACAAGGAGATGC R: CGACTGAAGAGCGAACCC	237
*Caspase-3*	NM_214131.1	F: TGGACTGTGGGATTGAGA R: ACCCGAGTAAGAATGTGC	214
*PI* *3* *K*	XM_013982221.1	F: ACGGAGGAGGTGCTCTGGAAC R: GGACTCGGGACTGGGCATCTC	150
*AKT1*	NM_001159776.1	F: GACGGCACCTTCATCGGCTAC R: CGCCACGGAGAAGTTGTTGAGG	81
*mTOR*	XM_003127584.6	F: GCACGTCAGCACCATCAACCTC R: GCCTCAGCCATTCCAACCAGTC	83
*β-actin*	NC_010445.4	F: CAAGGACCTCTACGCCAACAC R: TGGAGGCGCGATGATCTT	130

## Data Availability

The original contributions presented in this study are included in the article. Further inquiries can be directed to the corresponding authors.
